# Case report: If it is not asthma—think of lymphangioleiomyomatosis in younger female patients

**DOI:** 10.3389/fmed.2024.1328471

**Published:** 2024-02-12

**Authors:** Malene Helligsø Kirkeby, Elisabeth Bendstrup, Hanne Krogh Rose

**Affiliations:** ^1^Department of Oncology, Aarhus University Hospital, Aarhus, Denmark; ^2^Department of Respiratory Diseases and Allergy, Center for Rare Lung Diseases, Aarhus University Hospital, Aarhus, Denmark; ^3^Department of Clinical Medicine, Aarhus University, Aarhus, Denmark

**Keywords:** lymphangioleiomyomatosis, perivascular epithelial cell tumor, high-resolution computed tomography (HRCT), angiomyolipoma (AML), case report, asthma, mTOR inhibition (mTORi)

## Abstract

Lymphangioleiomyomatosis (LAM) is a rare lung disease predominantly affecting women, and it is characterized by the proliferation of smooth muscle cells and cystic lung destruction. LAM diagnosis is challenging due to varied clinical presentations and resemblance to common conditions such as asthma. We present two female cases where LAM was initially misdiagnosed. Case 1 describes a woman treated for asthma–chronic obstruction pulmonary disease overlap syndrome, while also undergoing treatment with vascular endothelial growth factor (VEGF) inhibitor pazopanib for a retroperitoneal leiomyoma, the latter responding well to treatment. Due to progressive dyspnea, pazopanib-induced pneumonitis was suspected. High-resolution computed tomography (HRCT) showed changes compatible with LAM. A revision of biopsies showed that the leiomyoma was in fact a lymphangioleiomyoma, and VEGF-D was increased. Both supported the LAM diagnosis. Treatment with mTORC1 inhibitor sirolimus was initiated. Case 2 describes a woman, who in resemblance with the woman from case 2 was also suspected of asthma and did not respond clinically to treatment. After several years, HRCT was performed and suspicion of LAM was raised. Transbronchial biopsy and later, an increased VEGF-D supported the LAM diagnosis. As in case 1, treatment with sirolimus was initiated. These cases underscore the importance of reevaluating diagnoses when treatments fail to yield expected results. Improved awareness and early detection of LAM can enhance patient outcomes and life quality. Early LAM diagnosis is vital as mTORC1 inhibitors such as sirolimus can prevent further decline in lung function. Notably, the response of case 2 to pazopanib treatment supports suggestions of its potential as a second-line therapy for perivascular epithelioid cell tumors (PEComas), including LAM.

## Introduction

Lymphangioleiomyomatosis (LAM) is a low-malignant rare disease of the lungs characterized by the proliferation of smooth muscle cells surrounding the lymphatics, blood vessels, and alveoli, and it leads to progressive cystic lung destruction and abdominal lymphangioleiomyomas (1). Half of LAM patients have angiomyolipomas (AML), which are often located in the kidneys (2). The classic clinical presentation of LAM includes progressive dyspnea, cough, and recurrent spontaneous pneumothorax, whereas chylothorax, chylous ascites, and hemoptysis are less common initial symptoms of LAM (2, 3). The extra-pulmonary symptoms of LAM may be abdominal distension and nausea due to lymphangioleiomyomas in the abdomen, pelvis, and retroperitoneum (4). LAM patients with renal AML may present with bleeding, which in some cases can be life-threatening (2).

LAM almost exclusively affects women, and the mean age of initial presentation is approximately 34 years. The prevalence is approximately 1/1.000.000, and up to 40% of all cases are associated with tuberous sclerosis complex (TSC) (2). The female sex hormones are important factors in LAM due to estrogen and progesterone receptors in LAM cells. This is of clinical importance because an accelerated decline in lung function can be seen during pregnancy or with exogenous estrogen use, and it also explains why the decline in lung function is more rapid in premenopausal women (3).

According to the American Thoracic Society/Japanese Respiratory Society LAM guidelines, a definite diagnosis of LAM requires one or more of the following factors in a patient who presents with clinical findings of LAM and characteristic findings in high-resolution computed tomography (HRCT): (1) tuberous sclerosis complex, (2) renal angiomyolipoma, (3) elevated serum VEGF-D ≥ 800 pg/mL, (4) chylous effusion, (5) lymphangioleiomyomas, (6) demonstrations of LAM cells or LAM cell clusters on cytological examination of effusions or lymph nodes, or (7) histopathological confirmation of LAM by lung biopsy or biopsy of retroperitoneal or pelvic masses (5).

Lymphangioleiomyomatosis can be medically treated with mTORC1 inhibitors such as sirolimus and everolimus. The end-stage disease may require lung transplantation (3).

In the following, two cases of LAM in young women are presented. In the first case, the medical presentation initially led to the diagnosis of a leiomyoma and asthma–chronic obstructive pulmonary disease (COPD) overlap syndrome. In the second case, the condition was initially interpreted as asthma. The aim of this case report is to emphasize the importance of recognizing features of rare conditions such as LAM, especially in cases where the initial symptoms can be quite common. Moreover, reevaluation of the initial diagnosis should be considered when therapeutic interventions are without the expected clinical effect.

## Case report

### Case 1

**Figure 1:**
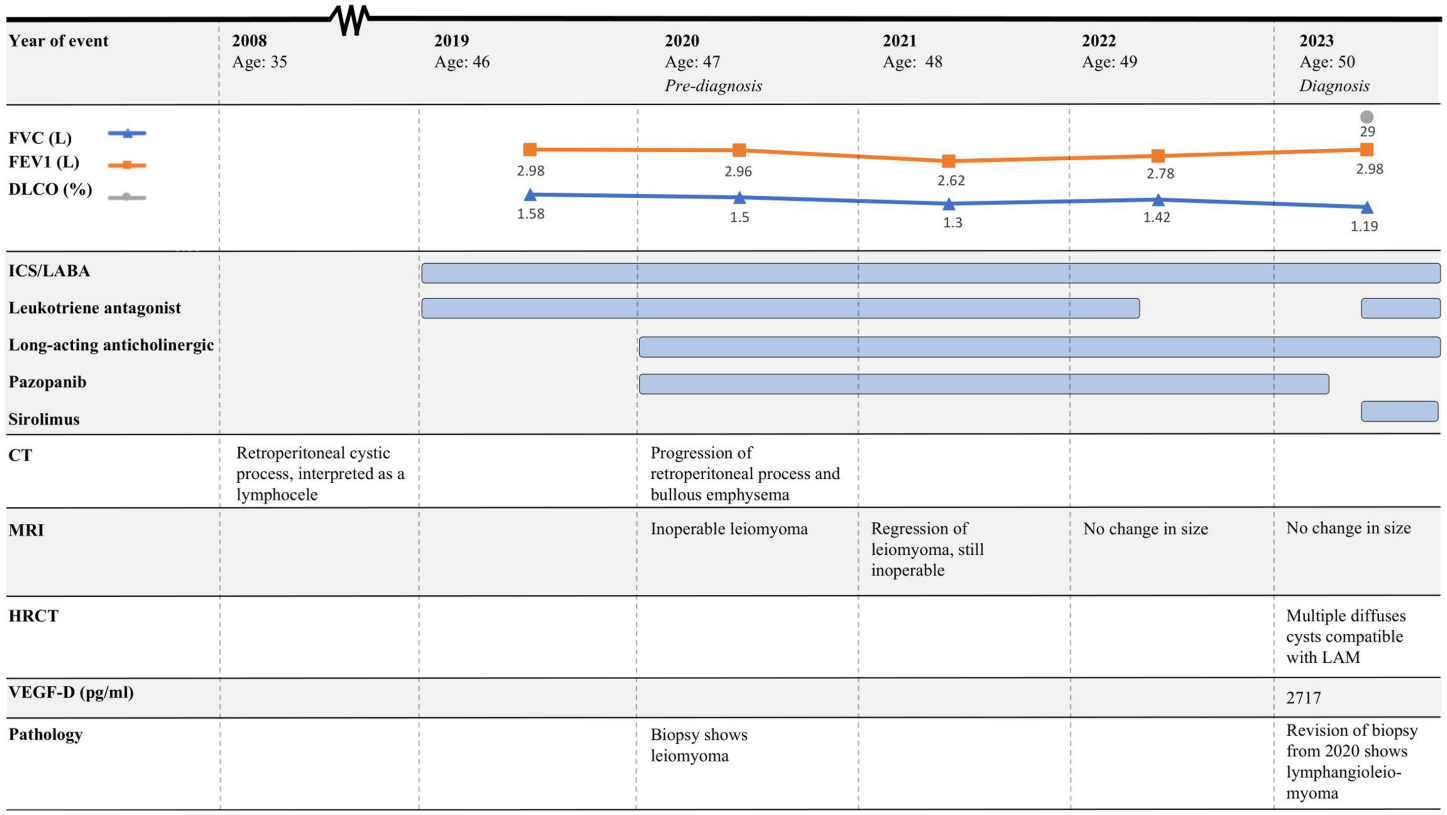
Timeline of case 1. CT, Computed tomography; MRI, Magnetic resonance imaging, HRCT, High-resolution computed tomography; FEV1, Forced expiratory volume in 1 s; FVC, Forced vital capacity; DLCO, Diffusion capacity for the lungs for carbon monoxide; LAM, Lymphangioleiomyomatosis; ICS, Inhaled corticosteroids; LABA, Long-acting beta-agonist; VEGFR, Vascular endothelial growth factor receptor.

The course of the disease is outlined in Figure 1. In 2019, a 46-year-old woman was brought to the Emergency Department due to coughing and dyspnea and was diagnosed with pneumonia. She was a smoker, and pulmonary function tests (PFTs) were obstructive (Figure 1). Therefore, underlying asthma–COPD overlap syndrome was suspected. Treatment with combined inhaled corticoid steroid (ICS) and long-acting beta-antagonist (LABA) was initiated, and she was referred for follow-up at the Asthma and Allergy Center at the Department of Respiratory Diseases and Allergy, where treatment was supplemented with a leukotriene antagonist. PFTs were still obstructive. At the time, she was perimenopausal with no history of hormone therapy. She had a 10-year-old child, and there was no record of respiratory symptoms during pregnancy and no additional pregnancies.

**Figure 2:**
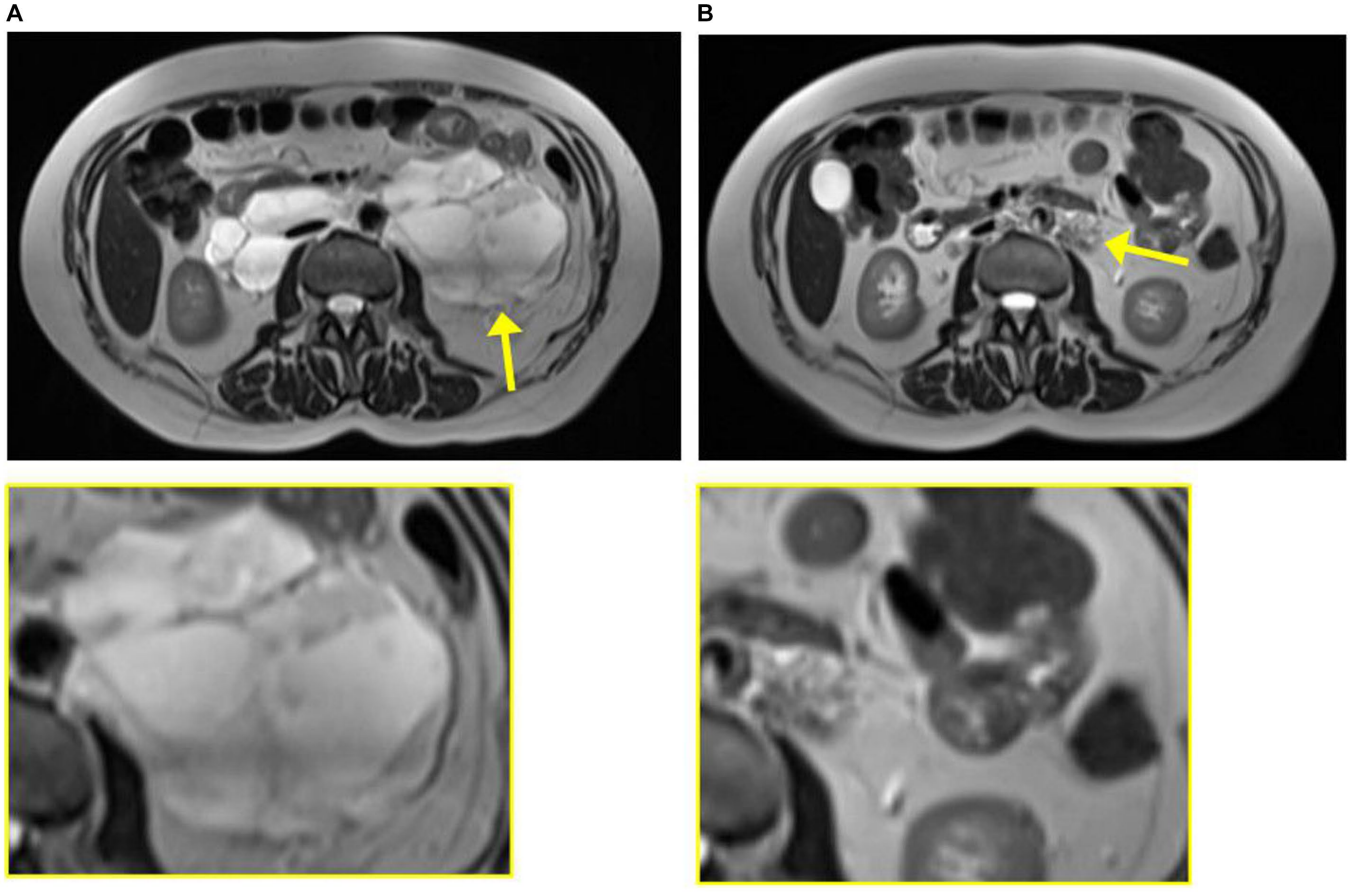
Magnetic resonance imaging from case 1 from 2020 at diagnosis of leiomyoma **(A)** and 2023 at diagnosis of lymphangioleiomyomatosis **(B)**. The tumor is indicated by the yellow arrow and enlarged in the yellow box.

In 2020, she was brought to the Emergency Department once again, this time presenting with abdominal pain, nausea, and vomiting. Computed tomography (CT) was performed, showing a retroperitoneal process and bullous emphysema. Several years before, in 2008, she had had a CT performed due to increased chromogranin A and suspicion of a neuroendocrine tumor. Back then, the same cystic process was seen and interpreted as a lymphocele. In 2020, CT was supplemented with magnetic resonance imaging (MRI), which raised suspicion of a benign leiomyoma infiltrating both the aorta and the inferior vena cava (Figure 2). A biopsy confirmed the diagnosis, and she was referred to the Oncology Department. Due to estrogen receptor positivity in the tumor, she was initially treated with an anti-estrogen. However, after 3 months, the tumor had not responded to treatment and the patient was experiencing progressive dyspnea. Due to the infiltrative nature of the tumor, the leiomyoma was inoperable and considered to be malignant by the treating oncologists. Treatment with anti-estrogen was discontinued, and treatment with the vascular endothelial growth factor receptor (VEGFR) inhibitor pazopanib was initiated. Concurrently, her asthma treatment was supplemented with long-acting anticholinergics.

The patient was regularly followed with an MRI, showing regression of the tumor (Figure 2). Each time, the scans were evaluated at a highly specialized gastrointestinal multidisciplinary conference, attended by oncologists, pathologists, gastrointestinal surgeons, and radiologists. Due to the infiltration of the large blood vessels, curative surgery was not an option. Clinically, she was experiencing increased abdominal pain despite tumor regression. For a period of 3 months in 2021, the treatment with pazopanib was put on pause, which resulted in tumor progression. Therefore, the treatment was resumed.

In 2022, follow-up related to her asthma–COPD overlap syndrome was concluded, and her health care was continued at her general practitioner. At the time, PFTs were still obstructive, but with an increase in forced expiratory volume in 1 s (FEV1) and forced vital capacity (FVC) (Figure 1).

**Figure 3:**
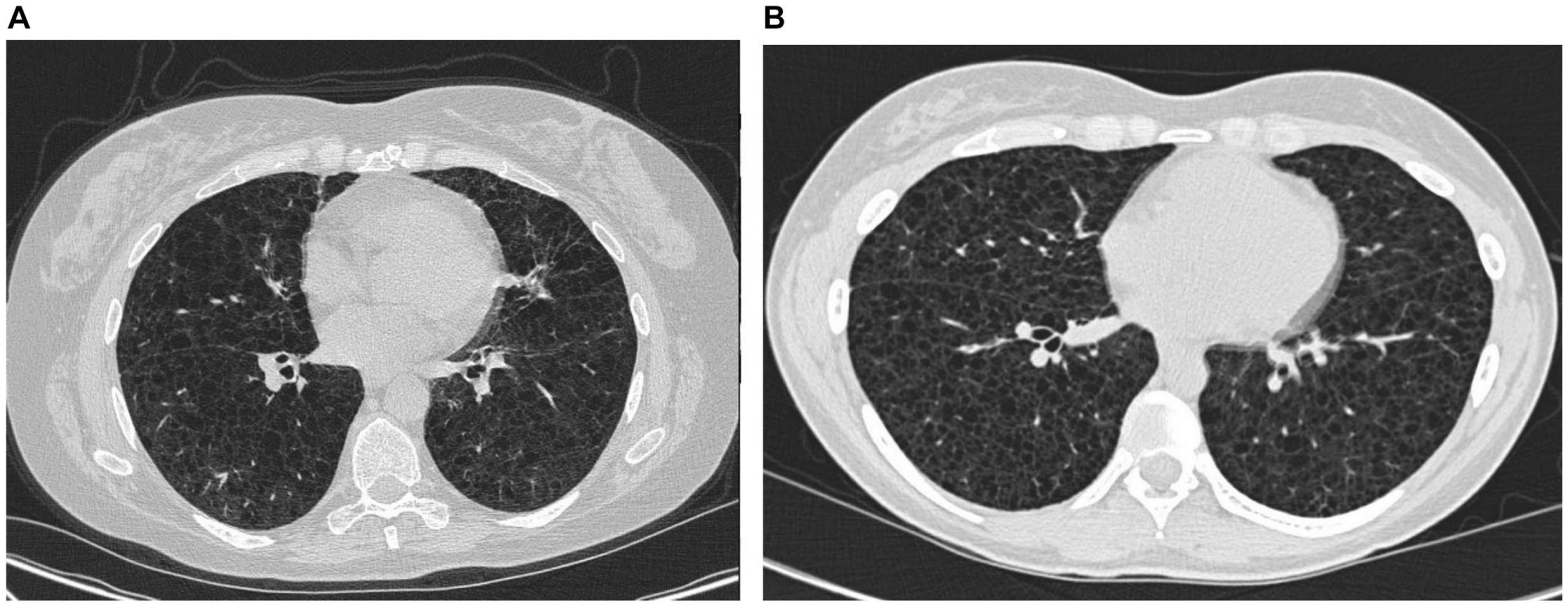
High-resolution computed tomography from case 1 **(A)** and case 2 **(B)**.

In February 2023, the patient experienced increasing breathlessness and was referred to an HRCT by the Oncology Department based on suspicion of treatment-induced pneumonitis due to ongoing treatment with pazopanib. The HRCT showed similar findings as before but this time interpreted as cysts and not emphysematous bullae (Figure 3A). In a multidisciplinary team discussion with thoracic radiologists and pulmonologists, the findings were found to be compatible with LAM. She was referred to the Center for Rare Lung Diseases at the Department of Respiratory Diseases and Allergy, where pathology re-evaluation of the retroperitoneal tumor was performed. It was initially evaluated as a leiomyoma with a predominance of smooth muscle cells. Upon second opinion, immunohistochemistry was highly positive for microphthalmia-associated transcription factor (MITF), muscle-specific actin, and human melanoma black 45 (HMB45) and negative for Melan-A, thus supporting a diagnosis of LAM. Later, the LAM diagnosis was supported by an elevated VEGF-D (Figure 1). She showed no other intra-abdominal features of LAM. Treatment with pazopanib was discontinued and treatment with sirolimus was initiated, supplemented by leukotriene antagonist and combined ICS/LABA/long-acting anticholinergics. Pulmonary function tests showed severe obstructive reduction and a low diffusion capacity for carbon monoxide (DLCO) (Figure 1). After 6 months of treatment with sirolimus, MRI showed no change in the size of the lymphangioleiomyoma.

In conclusion, the asthma–COPD overlap syndrome and the leiomyoma/lymphangioleiomyoma were both features of underlying LAM.

### Case 2

**Figure 4:**
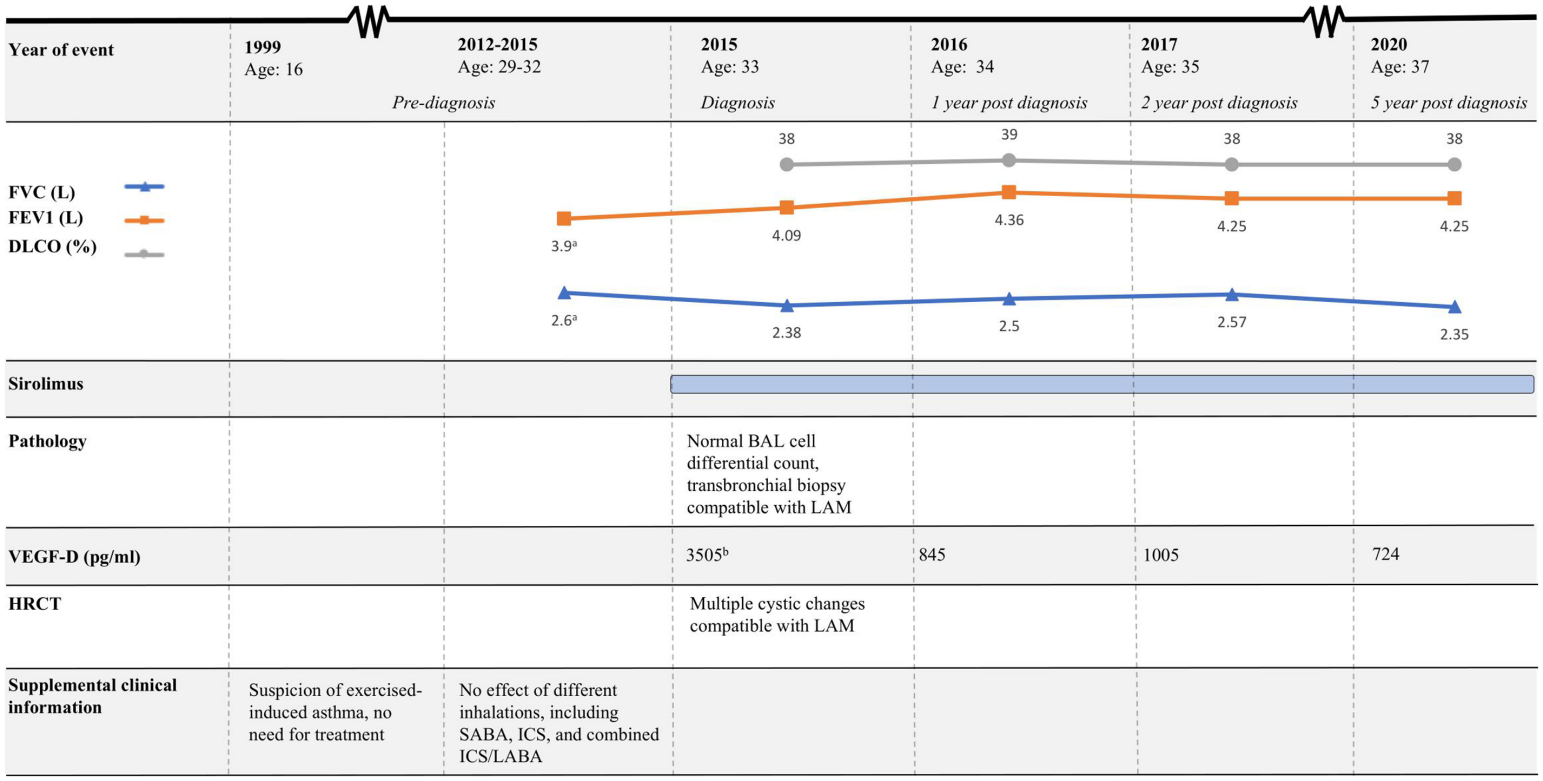
Timeline of case 2. HRCT, High-resolution computed tomography; FEV1, Forced expiratory volume in 1 s; FVC, Forced vital capacity; DLCO, Diffusion capacity for the lungs for carbon monoxide; LAM, Lymphangioleiomyomatosis; BAL, Bronchoalveolar lavage; SABA, Short-acting beta agonist; ICS, Inhaled corticosteroids; LABA, Long-acting beta-agonist. ^a^Pulmonary function tests performed in 2014. ^b^Result from VEGF-D analysis was available 4 months after BAL was performed.

The course of the disease is outlined in Figure 4. The second case describes a woman, who at the age of 16 was suspected of exercise-induced asthma, but the symptoms subsided without treatment. At age 29, she was diagnosed and treated for asthma at a private clinic. She was a non-smoker with no history of pregnancies or hormone therapy. Because she had had no clinical benefit from her asthma therapy, at age 32, she was referred to the Center for Rare Lung Diseases at the Department of Respiratory Diseases and Allergy in 2015. There, PFTs showed severe obstruction and DLCO reduction (Figure 4). An HRCT was performed showing multiple cysts compatible with LAM (Figure 3B). Cell differential count by bronchoalveolar lavage was normal, but a transbronchial biopsy showed a slight thickening of the alveolar septae with spindle-formed cells. By immunohistochemistry, these cells were positive for smooth muscle α-actin, muscle-specific actin, and estrogen receptors, and negative for Melan-A, thus compatible both morphologically and by immunohistochemistry with LAM. Later, a VEGF-D of 3,505 pg/mL supported the LAM diagnosis. Treatment with sirolimus was initiated. As indicated in Figure 4, her lung function has been stable since the initiation of the treatment. Due to skin changes, she was investigated for TSC at the Clinical Genetics Department, but this was disconfirmed.

In similarity to the first case, her asthma showed to be a feature of LAM.

## Discussion

While there is no universally agreed-upon global definition of a rare disease, a suggested threshold is approximately 50/100.000 (6). This indeed categorizes LAM as a rare disease, hence making the diagnosis of LAM, whether is it sporadic or related to TSC, challenging. The diagnosis is further complicated due to clinical variability and the presence of obstructive lung function, which often leads to initial misdiagnosis as asthma or COPD (3). In addition to mimicking obstructive lung diseases, LAM also belongs to a broader category of cystic lung diseases, which encompass various conditions characterized by the development of cystic spaces within the lung parenchyma. These diseases may have overlapping clinical presentations with LAM and include pulmonary Langerhans’ cell histiocytosis, Birt–Hogg–Dubé syndrome, lymphoid interstitial pneumonia, and amyloidosis (7). Due to the many differential diagnoses, comprehensive clinical, radiological, and histopathological assessments are essential to diagnose LAM. In addition, a family history of cystic lung disease and hamartomatous lesions in different organs including the skin is suggestive of TSC, and genetic testing should be performed in such patients (5).

In the two cases described above, respiratory symptoms were initially misinterpreted as asthma–COPD overlap syndrome and asthma, respectively. This led to a delayed diagnosis of LAM. The challenges associated with diagnosing LAM are particularly evident in case 1, where both CT and MRI were evaluated at highly specialized gastrointestinal multidisciplinary conferences. Despite this, LAM was not suspected. This oversight might be attributed to the rarity and limited awareness of the condition. Hence, the involvement of specialists in pulmonary medicine is essential for accurate LAM diagnosis. While it is understood that not all clinicians can be familiar with every medical condition, both cases underscore the importance of reevaluating initial diagnoses when therapeutic interventions fail to produce the expected clinical effect.

Moreover, case 2 emphasizes the importance of an early diagnosis and treatment initiation; the woman had a decline in lung function from the onset of respiratory symptoms until LAM diagnosis and treatment with sirolimus was initiated. It is important to note that mTORC1 inhibitors such as sirolimus only prevent further decline in lung function and do not restore lost lung function (3). Therefore, early detection of LAM is crucial for improving LAM patients’ quality of life and life expectancy. Due to the recent LAM diagnosis in case 1, it is premature to make similar conclusions.

Perivascular epithelioid cell tumors (PEComas) are a group of rare mesenchymal tumors originating from the perivascular epithelioid cell line. LAM is considered to be part of the PEComa family. There are no specific treatment guidelines for PEComas, but mTORC1 inhibitors such as sirolimus have proven effective. When it comes to second-line treatment, evidence is even weaker, but among others, VEGFR inhibitors such as pazopanib, sorafenib, and sunitinib are suggested as possible treatment options (8, 9). In the light of this, case 2 becomes rather interesting. There was significant regression of the lymphangioleiomyoma when treated with pazopanib, and the tumor further progressed in the 3-month-long treatment pause. This contributes to the list of case reports in which VEGFR inhibitors were effective in the treatment of PEComas such as LAM.

In conclusion, these case reports highlight the critical importance of identifying LAM in patients with respiratory symptoms, even when initial assessments suggest more common conditions such as asthma or COPD. The rarity and clinical variability of LAM can complicate diagnosis, leading to delays and adverse patient outcomes. The primary key learning point from the two cases is the necessity of reevaluating initial diagnoses when treatments prove ineffective. Furthermore, the response to pazopanib treatment in case 2 sheds light on potential second-line therapies for PEComas such as LAM, emphasizing the suggested effectiveness of VEGFR inhibitors.

## Patient perspective

In the first case, the patient naturally desired that a timely and accurate diagnosis be established in order to preserve pulmonary function. Nonetheless, she stated, “I understand and respect that it was very challenging for you to diagnose me, and I am just very pleased that you did so in the end.”

In the second case, the patient understandably experienced frustration due to the substantial duration between the onset of symptoms and the LAM diagnosis. Being diagnosed with a rare lung disease, previously unfamiliar to her, came as a surprise. However, she said that “finally having knowledge about my condition gives me great relief, and makes it possible for me to look ahead.”

## Data availability statement

The original contributions presented in the study are included in the article/supplementary material, further inquiries can be directed to the corresponding author.

## Ethics statement

Ethical approval was not required for the studies involving humans because formal ethical permission is not required for case stories. The studies were conducted in accordance with the local legislation and institutional requirements. The participants provided their written informed consent to participate in this study. Written informed consent was obtained from the individual(s) for the publication of any potentially identifiable images or data included in this article.

## Author contributions

MK: Writing – original draft. EB: Writing – review & editing. HR: Writing – review & editing.

## References

[1] Johnson S. Rare diseases. 1. Lymphangioleiomyomatosis: clinical features, management and basic mechanisms. Thorax. (1999) 54:254–64. doi: 10.1136/thx.54.3.254, PMID: 10325903 PMC1745441

[2] Johnson SR. Lymphangioleiomyomatosis. Eur Respir J. (2006) 27:1056–65. doi: 10.1183/09031936.06.00113303 16707400

[3] McCarthy C Gupta N Johnson SR Yu JJ McCormack FX. Lymphangioleiomyomatosis: pathogenesis, clinical features, diagnosis, and management. Lancet Respir Med. (2021) 9:1313–27. doi: 10.1016/S2213-2600(21)00228-9, PMID: 34461049

[4] Mavroudi M Zarogoulidis P Katsikogiannis N Tsakiridis K Huang H Sakkas A. Lymphangioleiomyomatosis: current and future. J Thorac Dis. (2013) 5:74–9. doi: 10.3978/j.issn.2072-1439.2013.01.03 23372952 PMC3548000

[5] Gupta N Finlay GA Kotloff RM Strange C Wilson KC Young LR. Lymphangioleiomyomatosis diagnosis and management: high-resolution chest computed tomography, transbronchial lung biopsy, and pleural disease management. An official American Thoracic Society/Japanese respiratory society clinical practice guideline. Am J Respir Crit Care Med. (2017) 196:1337–48. doi: 10.1164/rccm.201709-1965ST, PMID: 29140122 PMC5694834

[6] Richter T Nestler-Parr S Babela R Khan ZM Tesoro T Molsen E. Rare disease terminology and definitions-a systematic global review: report of the ISPOR rare disease special interest group. Value Health. (2015) 18:906–14. doi: 10.1016/j.jval.2015.05.008, PMID: 26409619

[7] Xu KF Lo BH. Lymphangioleiomyomatosis: differential diagnosis and optimal management. Ther Clin Risk Manag. (2014) 10:691–700. doi: 10.2147/TCRM.S50784 25187723 PMC4149398

[8] Czarnecka AM Skoczylas J Bartnik E Świtaj T Rutkowski P. Management strategies for adults with locally advanced, Unresectable or metastatic malignant perivascular epithelioid cell tumor (PEComa): challenges and solutions. Cancer Manag Res. (2023) 15:615–23. doi: 10.2147/CMAR.S351284, PMID: 37440783 PMC10335286

[9] Liapi A Mathevet P Herrera FG Hastir D Sarivalasis A. VEGFR inhibitors for uterine metastatic perivascular epithelioid tumors (PEComa) resistant to mTOR inhibitors. A case report and review of literature. Front Oncol. (2021) 11:641376. doi: 10.3389/fonc.2021.641376 33842348 PMC8032946

